# Naked-Eye 3-Dimensional Vision Training for Myopia Control: A Randomized Clinical Trial

**DOI:** 10.1001/jamapediatrics.2024.0578

**Published:** 2024-04-08

**Authors:** Rui Xie, Feng Zhao, Jianhong Yu, Bin Luo, Zhidong Jiang, Xiaoyun Qiu, Yingpin Cao, Yuxia Yang, Kezhe Chen, Yuan Zhang, Xiaoling Luo, Zhirong Wang, Yingting Zhu, Yehong Zhuo

**Affiliations:** 1 State Key Laboratory of Ophthalmology, Zhongshan Ophthalmic Center, Guangdong Provincial Key Laboratory of Ophthalmology Visual Science, Guangdong Provincial Clinical Research Center for Ocular Diseases, Sun Yat-sen University, Guangzhou, China; 2 Foshan Women and Children‘s Hospital, Foshan, Guangdong, China.; 3 Department of Ophthalmology, Shenzhen People‘s Hospital (The Second Clinical Medical College, Jinan University), Shenzhen, Guangdong, China

## Abstract

**Importance:**

Early onset of myopia increases the risk of high myopia, which can lead to irreversible retinal damage and even loss of central vision.

**Objective:**

To investigate the efficacy and safety of naked-eye 3-dimensional vision training (NVT) in preventing the progression of myopia in children.

**Design, Setting, and Participants:**

This randomized clinical trial was conducted in 3 hospitals from May 25, 2022, to February 24, 2023. Participants were children (aged 6-18 years) who had a diagnosis of myopia with a spherical equivalent refraction of −0.75 to −6.00 diopters (D).

**Intervention:**

Children in the intervention group received 20 minutes of NVT treatment every day, whereas children in the control group lived as usual without vision training.

**Main Outcome and Measure:**

The primary outcome was the change in axial length at 6 months. Spherical equivalent refraction (SER) was included as a secondary outcome.

**Results:**

Among 263 participants, 125 (47.5%) were male and 138 (52.5%) were female; the mean (SD) age was 10.3 (1.9) years (range, 6.1-15.6 years). A total of 227 patients (86.3%) completed the 6-month follow-up, including 102 in the intervention group and 125 in the control group. In the intervention group, the changes in axial length and SER at 6 months were 0.18 mm (95% CI, 0.16 to 0.20 mm) and −0.25 D (95% CI, −0.31 to −0.19 D), respectively. In the control group, the changes in axial length and SER at 6 months were 0.23 mm (95% CI, 0.21 to 0.25 mm) and −0.35 D (95% CI, −0.41 to −0.30 D), respectively. The differences in AL and SER between the 2 groups were significant (AL difference: −0.06 mm; 95% CI, −0.09 to −0.03; *P* < .001; SER difference: 0.10 D; 95% CI, 0.02 to 0.19; *P* = .02). No study-related adverse reactions were reported during follow-up.

**Conclusions and Relevance:**

NVT is a safe and promising means to control myopia progression in children with good adherence.

**Trial Registration:**

ClinicalTrials.gov Identifier: NCT05468775

## Key Points

**Question:**

Is naked-eye 3-dimensional (3-D) vision training effective in preventing myopia progression in children?

**Findings:**

In this randomized clinical trial that included 263 children, the change in axial length at 6 months was significantly different between the intervention and control group.

**Meaning:**

Naked-eye 3-D vision training is a promising means to control myopia progression in children.

## Teaser

This randomized clinical trial assesses the efficacy and safety of computer-based 3-dimensional vision training for preventing the progression of myopia in children.

## Introduction

Myopia is a common cause of visual impairment, and its prevalence continues to increase globally. According to current research results, it is expected that by 2050, 47.58 million people will experience myopia, accounting for about half of the world’s population.^1^ In Asia, especially in east Asia, the high incidence of myopia has become well known. In China, the high incidence of myopia among young people is a concern. It is predicted that the prevalence of myopia among students aged 6 to 18 years in China will be as high as 61.8% by 2030.^2^ Early onset of myopia increases the risk of high myopia, and complications of high myopia (such as a macular hole, optic neuropathy, etc) may lead to irreversible retinal damage and even loss of central vision, imposing a heavy burden on individuals, families, and society.^3,4^ The vision health problems of children and adolescents are related to the development of the country. Preventing and controlling myopia is urgent.

At present, the recognized causes of myopia can be roughly divided into 2 categories: genetic factors and environmental factors. Some studies have also found that the occurrence of myopia can be explained by peripheral defocus theory,^5^ form-deprivation theory,^6^ or accommodation theory.^7^ To prevent the occurrence and progression of myopia in children, many myopia intervention methods have been developed, including orthokeratology, soft contact lenses, and low-concentration atropine eyedrops. For interventions used in children and adolescents, safety is a key concern for clinicians and parents. Because of the adverse effects of drugs, it is still necessary to explore safer, noninvasive, and widely applicable myopia prevention and control methods.

Near work is one of the critical environmental factors that cause myopia. Due to the decrease in the adjustment response and the increase in the adjustment lag after a long period of close sight, the retina’s farsightedness is out of focus. Therefore, some scholars have proposed that adjustment lag may be the factor promoting the elongation of the eye axis and the progression of myopia, and improving the adjustment function through visual training may be an approach for myopia prevention and treatment.^8,9^ Some studies have shown that the use of optical, psychological, and other methods can reduce adjustment lag, increase adjustment ability, and improve visual comfort.^10,11^ Therefore, naked-eye 3-dimensional (3-D) vision training (NVT) is proposed as a feasible method. This study aimed to explore the effectiveness and safety of NVT for myopia prevention and control in children and adolescents.

## Methods

### Study Design

The Naked-Eye 3-D Vision Training Study is a hospital-based multicenter prospective randomized clinical trial that was conducted in southern China for 6 months, aiming to investigate the efficacy and safety of NVT in preventing the progression of myopia in children. Participants were randomly assigned to either the intervention group (receiving 20 minutes of computer-based NVT per day) or the control group (living as usual without receiving vision training). Ethical review approval was obtained from the medical ethics committee of Zhongshan Ophthalmic Center, Sun Yat-sen University (2021KYPJ193), Guangzhou, China, and the study was registered at ClinicalTrials.gov (NCT05468775). This prospective study followed the tenets of the Declaration of Helsinki. Only children whose legal guardians provided written informed consent were enrolled. We followed the Consolidated Standards of Reporting Trials (CONSORT) reporting guideline.

### Participants

We recruited participants from hospitals via posters or clinical optometrists. The inclusion criteria were (1) age 6 to 18 years, (2) diagnosis of myopia with a spherical equivalent refraction (SER) of −0.75 to −6.00 diopters (D), and (3) an understanding by participants and their legal guardians of the purpose of the study, as well as cooperation with treatment and related ocular examinations. The exclusion criteria were (1) astigmatism greater than 4.0 D; (2) anisometropia greater than 4.0 D; (3) best-corrected visual acuity less than 20/25; (4) other myopia control therapies in the last month; (5) ocular diseases such as congenital lens dislocation, congenital cataract, glaucoma, uveitis, microcornea, keratoconus, manifest strabismus, or other congenital ocular disorders; (6) systemic diseases such as nephrotic syndrome or diabetes; (7) allergy to compounds of tropicamide eyedrops; (8) poor cooperation during ocular examinations; or (9) a history of ocular surgery or structural changes in the eyeball caused by trauma. If the investigator determined that patients had contraindications or other conditions that would make them unsuitable to participate in the study, they were further excluded for reasons of safety or the patients’ best interest.

### Sample Size

Based on the results of previous studies,^12^ the annual (SD) axial elongation was assumed to be 0.50 (0.40) mm, with an intervention effect of 30%. A sample size of 226 participants (113 per group) achieved 80% power at a significance level of .05. Considering the loss to follow-up rate of 15%, the final sample size was 260 (130 in the intervention group and 130 in the control group).

### Intervention

**Figure 1.**
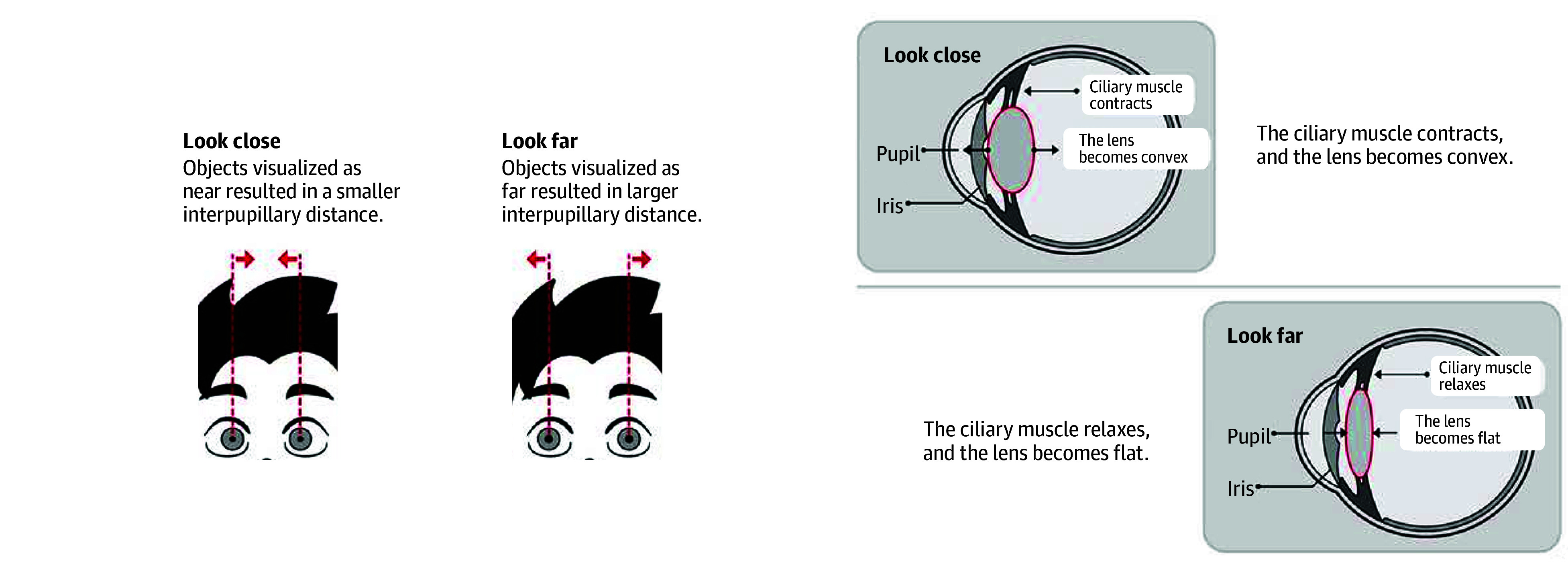
Change of the Eyes’ Position and Accommodation While Watching 3-Dimensional Images

During the 6 months of the trial, children in the intervention group were instructed to receive NVT (Figure 1 and Video) by viewing a computer program for 20 minutes per day, whereas participants in the control group lived as usual without receiving NVT. NVT intensity was varied at 12 levels, and each level of intensity involved 10 lessons, for a total of 120 lessons, to help users better adapt to the NVT images.

**Video.** Demonstration of Naked-Eye 3-Dimensional Vision Training Using naked-eye 3-dimensional display technology, children can see 3-dimensional, moving images without wearing any auxiliary glasses or equipment, thereby mobilizing eye movement and changing the state of eye adjustment, which may delay the progression of myopia.

Using an internet connection, the training details of the participants could be sent to their parents and the study coordinator. In this way, parents could monitor participants’ training in real time, and the study coordinator could remind parents to improve their child’s adherence if their child did not train for the expected amount of time. Training adherence was defined as effective training frequency / total training frequency × 100% (effective duration of training ≥15 minutes in a single day was recorded as 1 instance of effective training).

At 1, 3, and 6 months of the study, all participants were asked to return to the hospital for a follow-up examination to assess the progression of myopia and any adverse reactions. If participants or their legal guardians had any questions about the study, staff would be arranged to respond online. The number of participants at the 6-month visit was significantly affected by COVID-19 and the associated lockdown in the local city (eTable 3 in Supplement 2). In this situation, we provided reassurance and support in efforts to improve attendance at visits.

### Trial Outcomes

The primary outcome was the difference between the 2 groups in the change from baseline in axial length (AL) at 6 months. SER and binocular visual function were included as the secondary outcomes. Safety was assessed by the best-corrected visual acuity and uncorrected visual acuity in both groups. AL was measured using the IOL Master (Carl Zeiss 500; Meditec). Autorefraction was measured by an autorefractor (KR-8800, Topcon). At enrollment and the 6-month follow-up, autorefraction and subjective refraction were measured in the cycloplegic refractive state. Half of the cylindrical power plus the spherical power was used to calculate the SER. Visual acuity and binocular visual function tests were assessed by experienced optometrists.

### Statistical Analysis

Statistical analysis was performed using SPSS version 25.0 (IBM). Only the data of the right eye that met the enrollment criteria were used to represent participants because there was a strong correlation (*r* = 0.56-0.98) between the left and right eyes, according to Pearson coefficients. The left eye was used (n = 11) if the right eye failed to meet the inclusion criteria. Data were analyzed according to the intention-to-treat principle. As specified in the statistical analysis plan, the primary end point was analyzed with the use of an analysis of covariance (ANCOVA) model, with the baseline value as the covariate (trial protocol in Supplement 1). Other end points were summarized with the use of descriptive statistics and analyzed with an ANCOVA model as appropriate.

To test the robustness of the results, for the primary outcome, the changes from baseline measurements were also modeled with the use of a generalized linear mixed-effects model for repeated measures based on a participant-level analysis, which included fixed effects for baseline AL, age, sex, trial group, time, and the interaction trial group × time. Missing data were handled by multiple imputations with the use of the Markov chain Monte Carlo method for the primary and secondary efficacy end points that were assessed as continuous variables.

Subgroup analyses were conducted according to sex (male and female), age (6-10 years and 11-16 years), and baseline SER (≥−3.0 D and <−3.0 D). Efficacy was defined as the difference between the intervention group and the control group in AL change divided by the change in the control group. Data were presented as least-squares means with 95% CIs for continuous variables. The widths of the confidence intervals were not adjusted for multiple comparisons, so the intervals should not be used to infer definitive treatment effects for the secondary outcomes or subgroup analyses. A 2-sided *P* value of less than .05 was considered to indicate statistical significance for the primary outcome.

## Results

**Figure 2.**
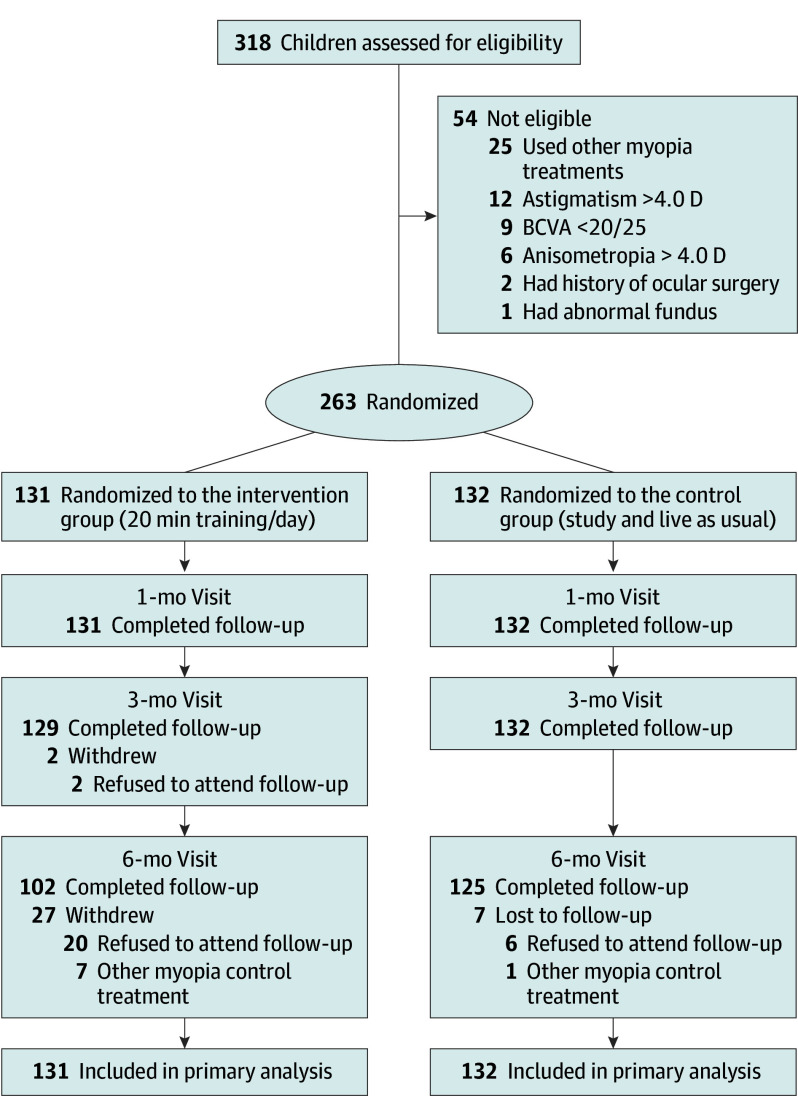
Flow of Participants in the Study BCVA indicate best-corrected visual acuity; D, diopter.

A total of 263 children were randomly assigned to either the NVT group (131 participants) or the control group (132 participants). Of the total number who underwent randomization, 227 participants (86.3%) completed the 6-month study (Figure 2), including 103 boys (45.4%) and 124 girls (54.6%). The mean (SD) age of participants was 10.3 (1.9) years (range, 6.1-15.6 years).

**Figure 3.**
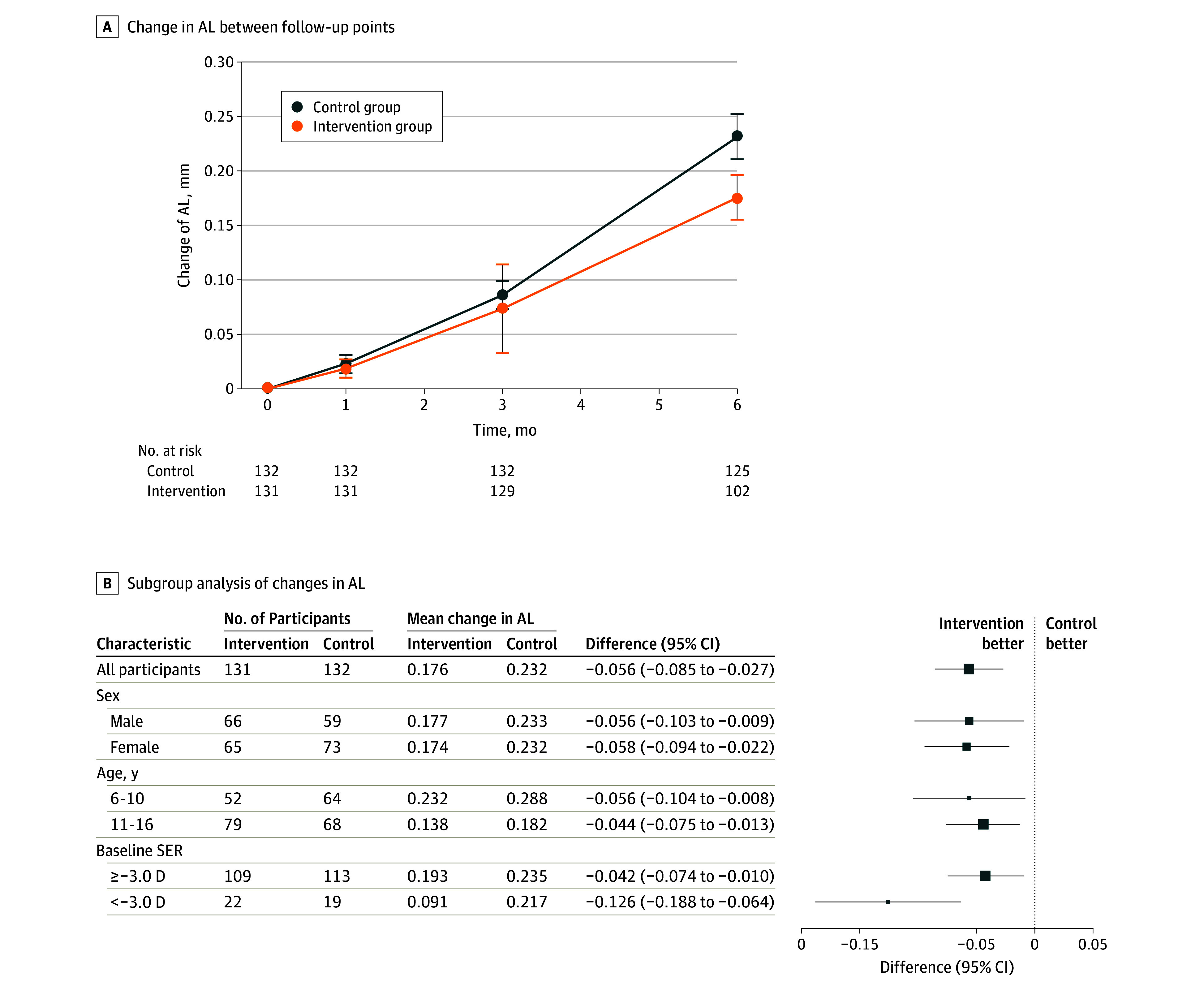
Axial Length (AL) Changes Between Follow-Up Points and a Subgroup Analysis SER indicates spherical equivalent refraction.

At baseline, the characteristics of the participants were similar in the 2 groups (Table 1). The mean (SD) AL was 24.35 (0.83) mm in the intervention group and 24.35 (0.82) mm in the control group. Over 6 months, the mean AL increased by 0.176 mm (95% CI, 0.155 to 0.197 mm) in the intervention group and by 0.232 mm (95% CI, 0.211 to 0.253 mm) in the control group (Figure 3). The difference (intervention − control) in the mean increase in AL was significant (−0.06 mm; 95% CI, −0.09 to −0.03; *P* < .001). For the SER, the difference (intervention − control) was 0.10 D (95% CI, 0.02-0.19; *P* = .02), representing a 29.1% reduction in myopia progression. The between-group differences in AL and SER increased over time (Table 2). At the 6-month follow-up, the changes of anterior chamber depth, white-to-white parameter, corneal power, and intraocular pressure in the intervention group and control group were 0.01 mm vs −0.05 mm, −0.06 mm vs −0.01 mm, −0.02 D vs −0.03 D, and 0.50 mm Hg vs 0.31 mm Hg, respectively.

**Table 1.** Comparison of Baseline Characteristics Between Groups

Variable	Mean (SD)	
	Intervention group (n = 131)	Control group (n = 132)
Sex, No. (%)		
Male	66 (50.4)	59 (44.7)
Female	65 (49.6)	73 (55.3)
Age, y	11.04 (1.96)	10.58 (1.96)
AL, mm	24.35 (0.83)	24.35 (0.82)
SER, D	2.08 (1.12)	1.98 (1.10)
UCVA	0.31 (0.20)	0.34 (0.20)
BCVA	1.00 (0.04)	1.00 (0.06)

Abbreviations: AL, axial length; BCVA, best-corrected visual acuity; SER, spherical equivalent refraction; UCVA, uncorrected visual acuity.

**Table 2.** Differences in Axial Length and Equivalent Spherical Diopter Between Groups

Follow-up	Mean (95% CI)		
	Intervention group	Control group	Difference
**AL change from baseline**			
1 mo	0.019 (0.011 to 0.027)	0.023 (0.015 to 0.031)	−0.004 (−0.015 to 0.008)
3 mo	0.074 (0.044 to 0.103)	0.086 (0.057 to 0.116)	−0.013 (−0.055 to 0.029)
6 mo	0.176 (0.155 to 0.196)	0.232 (0.212 to 0.253)	−0.056 (−0.085 to −0.027)
**SER change from baseline**			
1 mo	−0.058 (−0.090 to −0.027)	−0.080 (−0.111 to −0.048)	0.021 (−0.023 to 0.066)
3 mo	−0.117 (−0.162 to −0.073)	−0.158 (−0.203 to −0.114)	0.041 (−0.022 to 0.104)
6 mo	−0.251 (−0.311 to −0.191)	−0.354 (−0.414 to −0.295)	0.103 (0.019 to 0.188)

Abbreviations: AL, axial length; SER, spherical equivalent refraction.

Subgroup analysis was performed of the effect of myopia control (AL elongation) by different sexes, age groups, and baseline SER groups (eTable 1 in Supplement 2). For different sexes and different age groups, participants obtained similar effects from training. However, the difference in effect between the 2 baseline SER groups was statistically significant: children with a baseline SER less than −3.0 D benefitted more than those with a baseline SER of −3.0 D or more (−0.13 vs −0.04 mm, respectively; *P* = .04).

The efficacy of myopia control (AL and SER) increased as training adherence increased. With the adherence increasing from less than 50% to more than 75%, the efficacy increased from 26.16% to 40.08% in reducing axial elongation and from 22.28% to 42.39% in slowing SER progression. In addition, regardless of adherence, the rates of axial elongation and SER progression in the intervention group were slower than those in the control group.

No adverse reactions were reported during the study. A total of 29 participants (22.1%) discontinued the intervention, of whom 22 refused follow-up because of COVID-19 or other reasons, and 7 switched to other treatments. At the end of the study, all participants in the intervention group achieved a best-corrected visual acuity of 20/20 or better (eTable 2 in Supplement 2).

## Discussion

This randomized clinical trial aimed to explore the efficacy and safety of NVT as an intervention for preventing the progression of myopia in children. Compared with the control group, in the intervention group, NVT delayed myopia progression, and the effect became better as adherence improved. After 6 months, the mean AL progression was reduced by 35.86%, and the mean SER progression was reduced by 36.68%. No study-related adverse events were reported. Our findings provide solid evidence for NVT as an effective and safe intervention for preventing myopia progression.

NVT is an emerging method for myopia control. It is speculated that stereoscopic and moving visual icons promote the movement of children’s eyes, improve ciliary muscle spasms, and reduce the accommodative lag caused by long-term near work. The role of accommodative lag in the occurrence and development of myopia has been controversial. Some scholars believe that hyperopic defocus can lead to eye elongation, whereas accommodative lag will put the eye in the state of hyperopic defocus, so it is speculated that there is a close relationship between accommodative lag and myopia.^7,13^ Huang et al^8^ found that the immediate effects of viewing 3-D images include the reduction of accommodative lag and the increase of accommodative sensitivity. Accommodative flippers^9^ and virtual reality training^14^ were designed based on the principle of adjustment, but NVT has unique advantages, such as no additional wearing of eye equipment, a variety of content, and convenience. However, more research is needed to explore the potential mechanisms of NVT for myopia control.

Low-concentration atropine eyedrops and orthokeratology are the most common medication and optical interventions, respectively, for myopia control. The mechanism of low-concentration atropine may be related to inhibitory regulation, scleral remodeling, and increased UV exposure. Results from a classic study called LAMP (Low-Concentration Atropine for Myopia Progression) showed that using 0.01% atropine eyedrops for 1 year reduced SER progression by 27.16% and AL progression by 12.20%.^15^ By comparison, our intervention appeared to have a better effect on preventing myopia progression. In addition, atropine eyedrops will inevitably cause the user’s pupils to dilate, leading to adverse reactions such as photophobia. NVT may cause visual fatigue, dry eyes, and other discomfort, but this was not shown in the current stage of our study. The first randomized clinical study of orthokeratology for myopia control was conducted in Hong Kong. A total of 78 children completed 2 years of follow-up, and axial elongation was 43% slower in the intervention group. In the first, second, third, and fourth 6-month periods, the efficacy of myopic control was 55%, 32%, 29%, and 54%, respectively. The myopic control effect was reduced after the initial treatment period.^16^ Our study showed that NVT had an efficacy of 24.14% in axial control during the first 6-month period, but its efficacy over a longer duration needs further investigation.

In the subgroup analysis, we found differences in the efficacy of the intervention for children with different baseline SER values. For children with a baseline SER of −3.0 D or more, the intervention group achieved only 17.87% myopia control. Participants with a baseline SER less than −3.0 D achieved better efficacy: the AL elongation was 0.091 mm in the intervention group and 0.217 mm in the control group, achieving a myopia control efficacy of 58.06%. This may be because children with more severe myopia also had more severe accommodative lag, and the effect of NVT on improving accommodation was more pronounced. It could also be that these children were older and more adherent. We can design different training models for children of different ages and degrees of myopia and advocate training at the early stage of myopia to better control the progress of myopia.

Our findings suggested that myopia control effectiveness improves with adherence. Although this trend was not statistically significant, it still suggested that we need to improve participants’ adherence by enriching the training content and increasing interest to obtain better myopia control efficacy. During the 6-month follow-up, none of the participants reported study-related adverse events. At the end of the study, all participants in the intervention group achieved a best-corrected visual acuity of 20/20 or better. No fundus damage was found in participants. According to the available results, the myopia intervention method used in this study is safe.

### Limitations

There were some limitations to this study. First, the study lasted only 6 months, which was not enough to observe a complete myopia control effect. However, as the first study to use NVT for myopia control, this study opens the way for this myopia intervention and lays the foundation for future exploration of its long-term effects. Second, the study follow-up occurred during the most severe period of the COVID-19 epidemic in Guangdong Province. Even though we tried our best to improve the follow-up rate, there were still some children for whom follow-up was interrupted. At the same time, living and studying at home increased the time for near work, and the time for outdoor activities was greatly reduced, which may have accelerated the progress of myopia. Third, this study was only able to observe the effect of this intervention on myopia progression, and this result can only be generalized to the equipment and content used in this study. Further research is needed to explore the mechanisms and effects of different training intensities.

We considered the imbalance in the follow-up rate between the 2 groups for 2 reasons. First of all, compared with the control group, the intervention group not only needs to receive follow-up examinations on time but also needs to adhere to daily vision training. Some children in the intervention group expressed reluctance to continue training; although we tried to communicate and coordinate many times, the children still indicated that they would withdraw from the study and not continue the follow-up. Perhaps the training content was not interesting enough; to this point, we can further optimize it in the future and describe it as a current limitation. Second, the follow-up period of the study was the most severe period of the COVID-19 epidemic in Guangdong Province; mandatory home quarantine was directed in some places, and some children’s training had to be terminated. Despite our best efforts to improve follow-up rates, there were still some children whose follow-up was interrupted.

## Conclusions

The findings of this randomized clinical trial provide new evidence that NVT can reduce myopia progression without significant adverse effects. During 6 months of follow-up, NVT controlled axial elongation and SER progression. Additionally, the effectiveness of myopia control (AL and SER) increased as training adherence increased.

## Notes

Supplement 1. Trial protocol

Supplement 2. **eTable 1.** Relationship between myopia progression and training compliance **eTable 2.** Change of uncorrected visual acuity and best corrected visual acuity of participants **eTable 3.** Demographics and baseline ocular characteristics between participants with and without 6 month visit in the intervention and control group

Supplement 3. Data sharing statement
