# Supplementary material for: Naked-Eye 3-Dimensional Vision Training for Myopia Control: A Randomized Clinical Trial
Source: JAMA Pediatr. 2024 Apr 8;178(6):533–9. doi: 10.1001/jamapediatrics.2024.0578 (PMC11148688; doi:10.1001/jamapediatrics.2024.0578)
Supplement: Supplement 1. — Trial protocol [file jamapediatr-e240578-s001.pdf]

TRIAL PROTOCOL

Study Design

The Naked-Eye-3D Vision Training Study (NVTS) is a hospital-based multicenter prospective randomized controlled study in southern China, aiming to investigate the efficacy and safety of NVT in preventing the progression of myopia in children. The NVTS has obtained the ethical review approval number: 2021KYPJ193 (The Medical Ethics Committee of Zhongshan Ophthalmic Center, Sun Yat-sen University) and the clinical trial registration number: NCT05468775 (Website URL: ClinicalTrials.gov). This prospective study followed the tenets of the Declaration of Helsinki. Only children who were provided written informed consent for their legal guardians were enrolled. The participants of the intervention group adopted a one-year research insurance indemnity scheme, including compensation of up to RMB 100,000 for every serious adverse event no matter whether it is foreseeable or unforeseeable.

Subjects

The inclusion criteria were: (1) age: 6-18 years; (2) diagnosed as myopia with spherical equivalent refraction (SER) of −0.75 to −6.00 diopter (D); (3) participants and their legal guardians understood the purpose of this study and cooperated with treatment and related ocular examinations. The exclusion

criteria were: (1) astigmatism >4.0 D; (2) anisometropia >4.0 D; (3) best corrected visual acuity <20/25; (4) other myopia control therapies in the last month; (5) ocular diseases such as congenital lens dislocation, congenital cataract, glaucoma, uveitis, microcornea, keratoconus, manifest strabismus, or other congenital ocular disorders; (6) systemic diseases, such as nephrotic syndrome and diabetes; (7) allergy to compounds of tropicamide eye drops; (8) poor cooperation during ocular examinations; (9) a history of ocular surgery or structural changes in the eyeball caused by trauma. If the investigator determines that subjects have contraindications or other conditions that make them unsuitable to participate in this study, subjects will be further excluded for safety reasons or the interest of patients.

Sample Size

Based on the results of previous studies, the annual axial elongation was assumed to be 0.50 mm (standard deviation, 0.40 mm), with an intervention effect of 30%. A sample size of 226 participants (113 per group) achieved 80% power at a significance level of 0.05. Considering the loss to follow-up rate of 15%, the final sample size was 260 (130 in the intervention group and 130 in the control group).

Recruitment

Participants were recruited at Zhongshan Ophthalmic Center (Sun Yat-sen University, Guangzhou, China), Shenzhen People’s Hospital (Southern

University of Science and Technology, Shenzhen, China), Foshan Women and Children Hospital (Southern Medical University, Foshan, China). Advertisements placed in the hospital's public areas and recommendations from clinical optometrists were included in the recruitment strategy. 263 subjects were screened for inclusion in the baseline assessment.

Randomization

Participants were recruited by ophthalmologists and optometrists, and software was used by a clinical study coordinator to generate random numbers and randomly assign participants to the intervention group (receiving 20-min NVT per day) or the control group (living as usual without receiving vision training). Recruitment and grouping work were independent of each other, and the clinical study coordinator responsible for random-grouping work was not involved in the recruitment process to avoid subjective effects on the randomization process.

Study visits

Initial visits (recruitment, randomization, and baseline visit), as well as 1-, 3-, and 6-month follow-up visits, were included in NVTS. When participants or their legal guardians have any questions about the study, staff will be arranged to respond online. If participants develop a study-related adverse reaction, an unscheduled follow-up visit will be promptly arranged and clinicians will conduct professional evaluations for them. The axial length, visual acuity,

binocular visual function, and adverse reactions were collected at each follow-up visit.

Subject compliance

Each subject in the intervention group had training equipment. According to the data recorded by each training equipment, training compliance was defined as effective training frequency / total training frequency * 100% (effective duration of training ≥ 15 minutes in a single day was recorded as one instance of effective training).

Naked-eye-3D vision training

In this study, mobile and portable devices were provided by Holo See Medical Technology Co., Ltd. (Shenzhen, China), which were used at home by participants in the intervention group. The device is equipped with a pupil-tracking camera. After connecting to the Internet, the training details of participants can be sent to the study coordinator. At the same time, parents can also check their children’s training in real time through the mobile application. In this way, parents can assist the study coordinator to monitor and improve the compliance of participants.

The device has obtained the national compulsory product certification (certificate number: 2021200805000421) and has passed the international standard IEC62471 blue light hazard test, with no known damage that can be caused. Due to the concern that some users may have a weak stereoscopic

vision ability and feel dizzy at the beginning of use, all the children were asked to try it during the recruitment stage, but no one showed any symptoms of discomfort. Meanwhile, to help participants better adapt to the training imaging, the NVT intensity was varied at 12 levels. Positive and negative parallaxes gradually increased from 0% to >40%. Positive parallax means the intersection is behind the screen. Negative parallax means the lines of vision of the two eyes intersect in front of the screen. The intensity is defined as the distance between the intersection and the screen divided by the distance between the screen and the eye, expressed as a percentage. The ideal distance between the device screen and the eye is 50 cm. Each level of intensity has ten lessons, a total of 120 lessons. The participants of intervention group work out two lessons a day, and the intensity increases by one level every 5 days. Attention was also paid to the feedback of participants and their parents, monitoring them for any adverse reactions related to the study.

Distance visual acuity

Distance visual acuity was measured with and without spectacles for all participants, by a logarithmic visual acuity chart (Precision Vision, La Salle, IL, USA) used at a distance of 4 m. The subjective refraction was conducted to obtain the best corrected visual acuity for those with distance visual acuity <20/20. Snellen acuity was shifted to the LogMAR scale for enrollment criteria and statistical analysis.

Slit-lamp examination

Ophthalmologists conducted slit-lamp biomicroscopy (Topcon, PS-62E, Japan) before cycloplegia, examining anterior and posterior segments at the slit lamp and recording any abnormalities (such as iris abnormalities, corneal disease) in the anterior segment.

Cycloplegic autorefraction

Cycloplegic autorefraction was only performed at the enrollment and the sixth-month follow-up. Refraction data for each eye were measured three times using an autorefractor (KR-8800, Topcon, Tokyo, Japan) and averaged to the desired accuracy (i.e., axis < 5 degrees, spherical and cylindrical power < 0.25 D); otherwise, the whole measurement was repeated. Compound tropicamide (0.5% phenylephrine hydrochloride + 0.5% tropicamide) was used to induce cycloplegia. Two drops were given for each eye at 10-minute intervals for three times. Ten minutes after the third application, two experienced ophthalmologists double-checked patients to make sure sufficient cycloplegic conditions: a pupil diameter over 6 mm and no light reflex. Half of the cylindrical power plus the spherical power was used to calculate SER.

Subjective refraction

At the enrollment and the sixth-month follow-up, subjective refraction was performed in the cycloplegic refractive state. The first was to conduct streak retinoscopy, followed by using a letter chart and a phoropter for subjective

refraction. The optimal visual performance and patient comfort were then ensured through the binocular refinement and balancing of refractive correction. The endpoint criterion was a maximum plus sphere and minimum minus cylinder consistent with best corrected visual acuity.

Ocular biometric measurement

The AL, anterior chamber depth (ACD), corneal power (CP) and white-to-white parameter (WTW) were measured using the IOL Master (Carl Zeiss 500, Meditec, Oberkochen, Germany). Before cycloplegia, five repeated measurements were conducted and averaged.

Optical coherence tomography (OCT)

Choroidal thickness is an optional outcome for participants, as measured by OCT (Cirrus HD-OCT 5000, Carl Zeiss Meditec, Dublin, CA, USA). Cirrus HD-OCT 5000 has a scanning speed of 27,000 times per second at a laser wavelength of 840 nm, generating an axial resolution of 5 μm and a scanning depth of 2 mm. The subject’s macular area was scanned using HD Cross or HD 5 Line Raster in the EDI mode. Each image covered a 6 x 6 mm area centered on the fovea. Choroid thickness is defined as the distance between the retinal pigment epithelium-Bruch’s complex and the outer choroid-scleral margin, as measured manually by an experienced physician. The final thickness was calculated as the arithmetic mean of the three measures. In the

OCT examination, one patient was found to have macular abnormalities, who was therefore not included in this study.

Accommodative parameters

In this study, an inspection of accommodative parameters was designed to exclude abnormalities in binocular vision, including accommodative response, accommodative facility, amplitude of accommodation, and near point of convergence. After excluding any measurements contaminated by external eye movements, five readings were conducted and averaged.

Primary outcome

The primary outcome was to investigate whether NVT is effective and safe in preventing the progression of myopia in children by assessing the axial length elongation in both groups.

Secondary outcome

Measurements other than axial length were considered the secondary outcome.

Data collection, management, and monitoring

All researchers received training in clinical protocol and data management before the study implementation.

According to the study protocol and medical records, the case report form (CRF) was designed by researchers. Measurement results and symptoms of

discomfort at baseline and each follow-up were collected and recorded on the CRF. Training data (time point, duration) of subjects in the intervention group were recorded in the training equipment, which could be recorded and reviewed from the cloud. The medical records of participants were securely stored, to which only investigators and monitors had access. Data and information of participants were not used to support other studies without the consent of subjects and their guardians. In the process of data analysis, the identity information of participants was hidden to protect their privacy.

Participants were allowed to withdraw from the study at any time and for any reason. Researchers could also withdraw participants from the study to protect their safety.

To ensure the quality of this clinical study and avoid subjective effects on the randomization process, the staff responsible for recruitment and grouping were not involved in each other’s work. At each study site, ethics committees could monitor the progress and safety of the study at any time and were independent of researchers in the study.

STATISTICAL ANALYSIS PLAN

Statistical analysis will be performed using IBM SPSS Statistics (version 25.0; IBM Corp., Armonk, NY, USA). Only the data from the right eye that met the enrollment criteria will be used to represent participants because there is a strong correlation between the left and right eyes. The left eye will be used if the right eye fails to meet the inclusion criteria. Data will be analyzed according to the intention-to-treat principle. The primary endpoint will be analyzed with the use of an analysis of covariance (ANCOVA) model, with the baseline value as the covariate, or other appropriate analysis methods. Other endpoints will be summarized with the use of descriptive statistics and analyzed with an ANCOVA model as appropriate. Missing data will be handled by multiple imputations with the use of the Markov chain Monte Carlo method for the primary and secondary efficacy endpoints that were assessed as continuous variables. Subgroup analyses were conducted according to sex, age and baseline SER. Data were presented as least squares means with 95% confidence intervals for continuous variables. A two-sided P value of less than 0.05 was considered to indicate statistical significance for the primary outcome.
