# Supplementary material for: Naked-Eye 3-Dimensional Vision Training for Myopia Control: A Randomized Clinical Trial
Source: JAMA Pediatr. 2024 Apr 8;178(6):533–9. doi: 10.1001/jamapediatrics.2024.0578 (PMC11148688; doi:10.1001/jamapediatrics.2024.0578)
Supplement: Supplement 2. — eTable 1. Relationship between myopia progression and training compliance eTable 2. Change of uncorrected visual acuity and best corrected visual acuity of participants eTable 3. Demographics and baseline ocular characteristics between participants with and without 6 month visit in the intervention and control group [file jamapediatr-e240578-s002.pdf]

Supplementary Online Content

Xie R, Zhao F, Yu J, et al. Naked-eye 3-dimensional vision training for myopia control: a randomized clinical trial. *JAMA Pediatr*. Published online April 8, 2024. doi:10.1001/jamapediatrics.2024.0578

eTable 1. Relationship between myopia progression and training compliance

eTable 2. Change of uncorrected visual acuity and best corrected visual acuity of participants

eTable 3. Demographics and baseline ocular characteristics between participants with and without 6-month visit in the intervention and control group

This supplementary material has been provided by the authors to give readers additional information about their work.

© 2024 Xie R et al. *JAMA Pediatrics.*

eTable 1. Relationship between myopia progression and training compliance

Treatment compliance	No.	Mean (SD)
**Change of AL**		
Intervention group total	102	0.152 (0.091)
<50%	24	0.175 (0.100)
50%~75%	32	0.149 (0.073)
>75%	46	0.142 (0.097)
Control group	125	0.237 (0.123)
**Change of SER**		
Intervention group total	102	−0.233 (0.255)
<50%	24	−0.286 (0.229)
50%~75%	32	−0.223 (0.241)
>75%	46	−0.212 (0.277)
Control group	125	−0.368 (0.364)
AL = axial length; SER = spherical equivalent refraction; SD = standard deviation.		

© 2024 Xie R et al. *JAMA Pediatrics.*

eTable 2. Change of uncorrected visual acuity and best corrected visual acuity of participants

Variable	N (%)	
	Intervention group	Control group
**Change of UCVA**		
≥2 worse	22 (22.22)	35 (28.93)
±1	54 (54.55)	78 (64.46)
≥2 improve	23 (23.23)	8 (6.61)
**BCVA**		
<20/20	0 (0)	1 (0.80)
≥20/20	102 (1)	124 (99.20)
UCVA = uncorrected visual acuity; BCVA = best corrected visual acuity.		

© 2024 Xie R et al. *JAMA Pediatrics.*

eTable 3. Demographics and baseline ocular characteristics between participants with and without 6 month visit in the intervention and control group

Variable	Intervention group (N=131)		Control group (N=132)	
	without 6 month visit (N=29)	with 6 month visit (N=102)	without 6 month visit (N=7)	with 6 month visit (N=125)
**Gender, N (%)**				
Male	19 (65.52)	47 (46.08)	3 (42.86)	56 (44.80)
Female	10 (34.48)	55 (53.92)	4 (57.14)	69 (55.20)
**Age, Mean (SD)**	9.59 (1.32)	11.46 (1.92)	12.43 (1.51)	10.50 (1.98)
**AL, Mean (SD)**	23.96 (0.79)	24.46 (0.82)	24.37 (0.57)	24.35 (0.83)
**SER, Mean (SD)**	−1.83 (0.74)	−2.15 (1.20)	−1.54 (0.91)	−2.01 (1.11)
**UCVA, Mean (SD)**	0.34 (0.18)	0.30 (0.21)	0.48 (0.29)	0.33 (0.19)
**BCVA, Mean (SD)**	0.99 (0.03)	1.00 (0.05)	1.00 (0.00)	1.00 (0.06)

SD = standard deviation; AL = axial length; SER = spherical equivalent refraction; UCVA = uncorrected visual acuity; BCVA = best corrected visual acuity.

© 2024 Xie R et al. *JAMA Pediatrics.*
