# Supplementary material for: Naked-Eye 3-Dimensional Vision Training for Myopia Control: A Randomized Clinical Trial
Source: JAMA Pediatr. 2024 Apr 8;178(6):533–9. doi: 10.1001/jamapediatrics.2024.0578 (PMC11148688; doi:10.1001/jamapediatrics.2024.0578)
Supplement: Supplement 3. — Data sharing statement [file jamapediatr-e240578-s003.pdf]

Data Sharing Statement

Xie. Naked-Eye 3-Dimensional Vision Training for Myopia Control. *JAMA Pediatr*. Published April 08, 2024. doi:10.1001/jamapediatrics.2024.0578

Data
Data available: No
